# Multifaceted progressive neurotuberculosis in a single patient: from miliary tuberculomas to cortical venous infarct

**DOI:** 10.1259/bjrcr.20180020

**Published:** 2018-07-12

**Authors:** Nivedita Agarwal, Lorenza Lenzi, Nicoletta Dorigoni, Susanna Cozzio, Sabino Walter Della Sala

**Affiliations:** 1 Section of Radiology, Santa Maria del Carmine Hospital, Rovereto, Italy; 2 Center for Mind/Brain Sciences, University of Trento, Rovereto, Italy; 3 Department of Radiological Sciences, Section of Neuroradiology, University of Utah, Salt Lake City, UT, USA; 4 Section of Internal Medicine, Santa Maria del Carmine Hospital, Rovereto, Italy; 5 Section of Infectious Diseases, Santa Chiara Hospital, Trento, Italy

## Abstract

Neurotuberculosis is a potentially fatal disease which requires prompt diagnosis and immediate multidrug antitubercular treatment as per international guidelines. There is evidence that the bacterial spread can continue even during therapy at least in its initial stages. We monitored our patient not only with chest X-rays but with brain MRI during the first 6 weeks. To our surprise on serial MRI, during treatment, we found several new localization of the disease in a pauci-symptomatic patient. These included vessel wall inflammation (vasculitis), arachnoiditis and hypophysitis. At 4 weeks of treatment, the patient complained of dizziness and vomiting which were first dismissed as treatment side-effects but MRI revealed multiple cortical venous hemorrhagic infarcts. We report this case to emphasize the importance of neuroimaging even in case of the most subtle symptoms and that disease can continue to progress in the initial phase of treatment which may require additional therapeutic intervention.

## Introduction

Nneurotuberculosis is a potentially fatal disease which requires prompt diagnosis and immediate multidrug antitubercular treatment as per international guidelines. There is evidence that the bacterial spread can continue even during therapy at least in its initial stages. Methods: we monitored our patient not only with chest X-rays and with brain MRI during the first 6 weeks. Resuts: to our surprise on serial MRI, during treatment, we found several new localization of the disease in a pauci-symptomatic patient. These included vessel wall inflammation (vasculitis), arachnoiditis and hypophysitis. At 4 weeks of treatment, the patient complained of dizziness and vomiting which were first dismissed as treatment side-effects but MRI revealed multiple cortical venous hemorrhagic infarcts. Conclusion and Advances in Knowledge: we report this case to emphasize the importance of neuroimaging even in case of the most subtle symptoms and that disease can continue to progress in the initial phase of treatment which may require additional therapeutic intervention.

## Clinical presentation

A 36-year-old female from Pakistan living in Italy for the past 10 years presented to the emergency room with chest pain and dyspnea. She also complained of vertigo, headache, and apathy which were subtle and could not be well investigated due to language barrier. Her neurological examination was otherwise negative for focal localization. Her past history revealed fine needle aspiration of a submandibular lymph node 2 years ago and again 1 month before hospital admission. In both occasions histological analysis showed small monomorphic lymphocytes and macrophages and were considered non-specific and no further diagnostic tests were undertaken. A chest X-ray was never acquired.

## Differential diagnosis

Our patient presented with non-specific neurological symptoms (vertigo, headache, and apathy) with pulmonary tuberculosis . It can be debated whether these symptoms are enough to order a brain CT and/or a brain MR. However, international guidelines require that if there is any evidence of pulmonary miliary disease, then the patient should be treated as having a systemic disease and any subtle neurological symptoms calls for neuroimaging.^1^ MRI is highly sensitive with respect to CT in defining lesions of the basal ganglia, midbrain, and brainstem and for evaluating all forms of suspected spinal tuberculosis. Multiple miliary lesions in the brain parenchyma with meningeal enhancement over the optic nerves carries the following differential diagnosis:

- Fungal disease: cryptococcus, brucellosis, neurocysticercosis, toxoplasmosis.
- Space occupying lesions: lymphoma, metastasis (primary neuroectoderm tumors, melanoma).

Epidemiologic factors, patient age, HIV infection, and immunologic status will help in narrowing the differential diagnosis.

## Investigations

**Figure 1.**
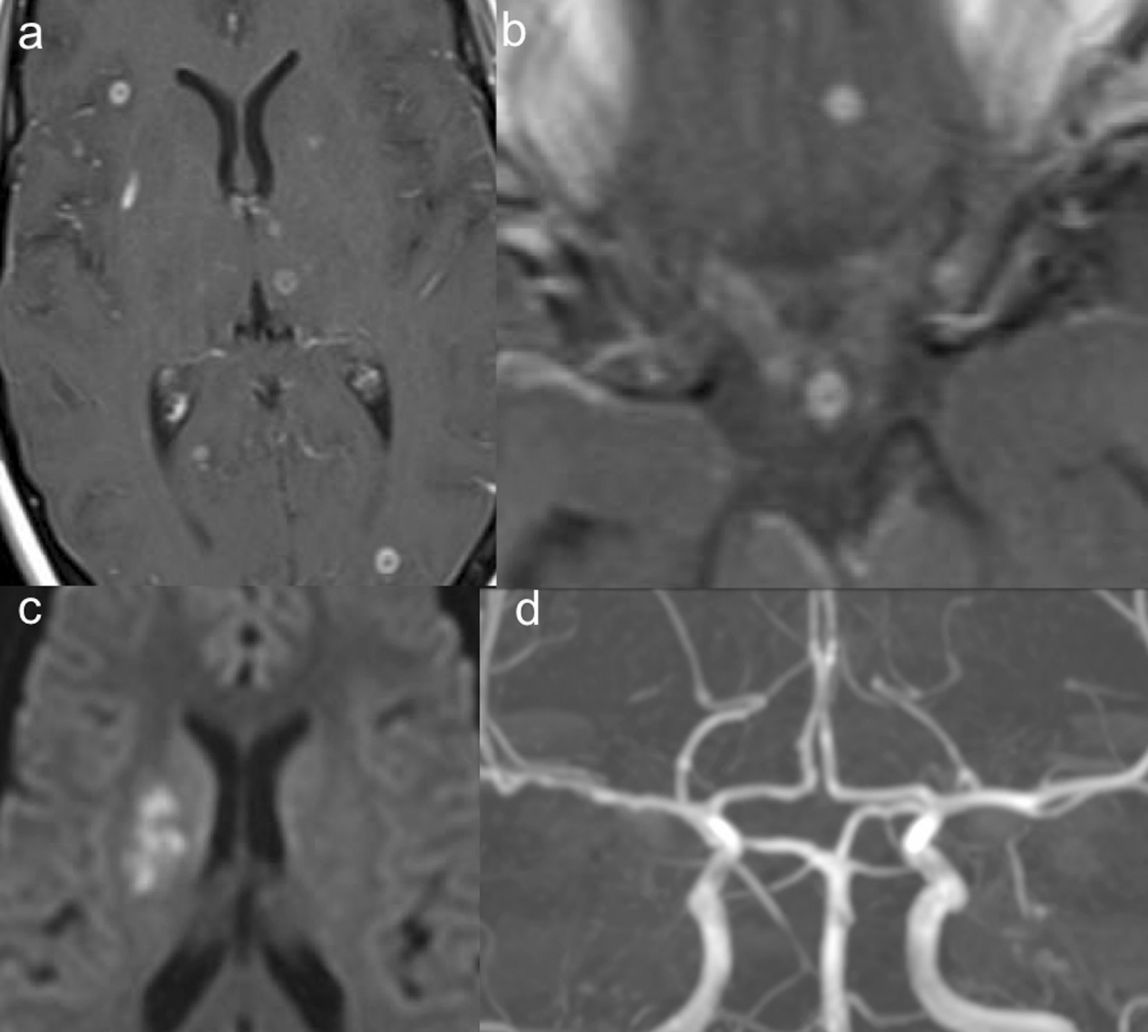
(a) T1 post-gadolinium shows multiple contrast-enhancing tuberculomas; (b) T1 post-gadolinium images show leptomeningeal enhancement and optic nerve sheath nodular enhancement; (c) Diffusion-weighted images showing hyperintense lesion in the right putamen corresponding to ischemic infarct; and (d) Time-of-flight shows wall irregularity of the right M1 segment.

**Figure 2.**
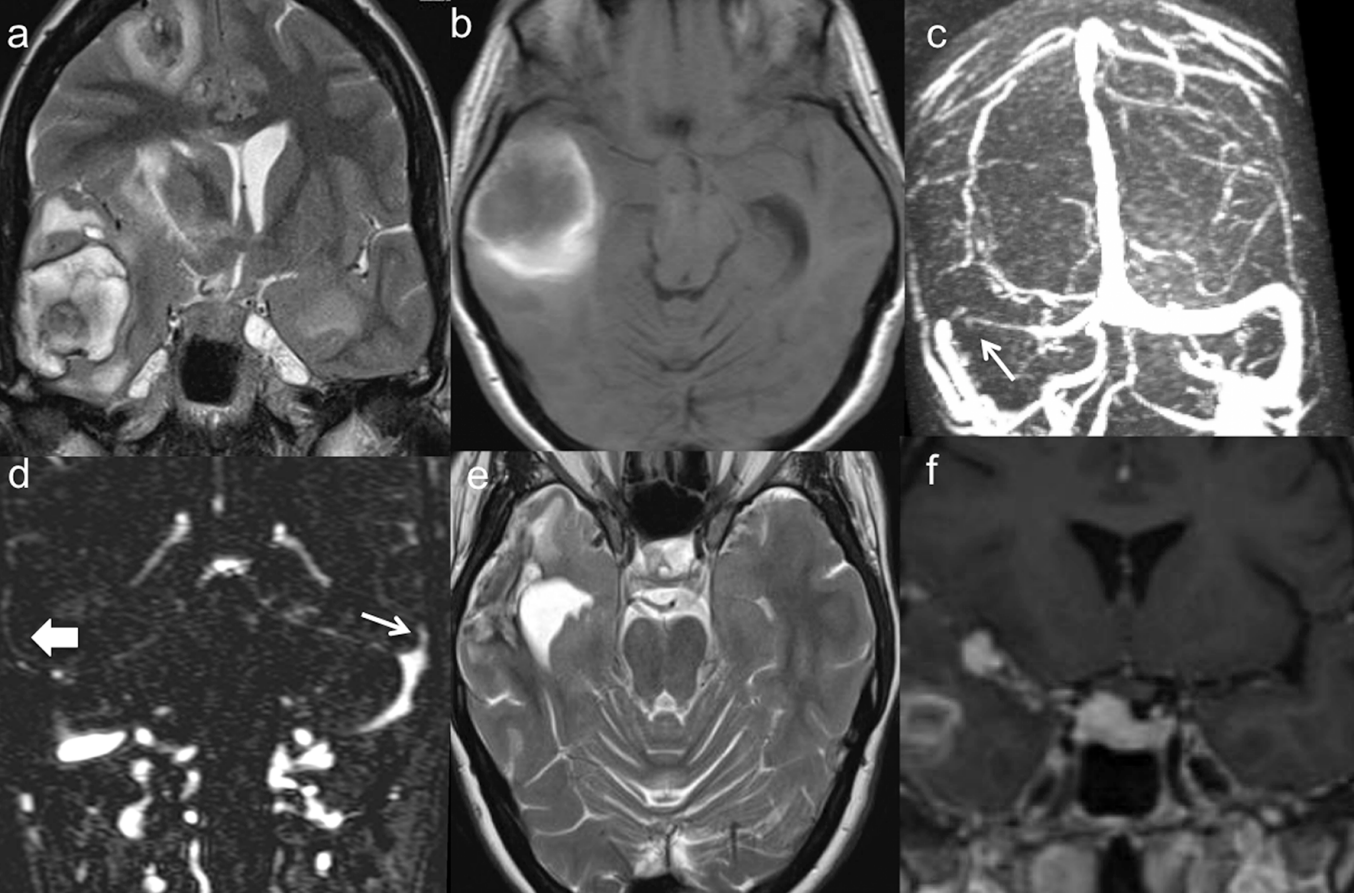
(a) Voluminous hemorrhagic infarct characterizedby heterogenous signal in T2-weighted images is seen at the level of the right temporal lobe with midline shift; (b) Hemorrhagic infarct as seen on T1-weighted image, (c) Narrowing of multipledural venous sinuses; (d) thinning of cortical veins; (e) resolution of the venous infarct with anticoagulant therapy; and (f) New enhancing mass in the sellar and parasellar region identifies a granulomatous solid hypophyseallesion.

A chest X-ray was performed that showed mild interstitial infiltrate, focal multiple opacities with a miliary distribution and consolidation of the right basal lobe, confirmed with a high resolution CT. Gene amplification on Bronchoalveolar lavage (BAL) was positive for *M. tuberculosis* susceptible to rifampicin, streptomycin, isoniazide, ethambutol, and pyranizamide. A diagnosis of pulmonary TB was made and medical treatment with four-drug antitubercular therapy was immediately started. Since she presented with subtle neurological symptoms, a brain MRI was requested. To our surprise, multiple tuberculomas with a miliary distribution were seen in both the supratentorial and the infratentorial brain parenchyma (Figure 1a). In addition to tuberculomas, nodular enhancement was seen along the prechiasmatic optic nerve sheaths bilaterally (Figure 1b). Focal leptomeningeal enhancement was seen in the right sylvian fissure (Figure 1b). The patient was now diagnosed with CNS TB and TB meningitis (TBM) prompting us to include dexamethasone to the treatment regimen as per international guidelines.^2^ While clinically stable a follow-up brain MRI was performed at 2 weeks that showed a global decrease in the number of tuberculomas and a reduction in perilesional edema. However, an arterial arterial ischemic infarct in the right putamen was identified characterized by increased signal of trace image (Figure 1c) and relatively hypointense signal on ADC. Her neurological examination was negative. On time-of-flight MR irregular wall and multiple stenosis was observed in the right M1 segment of the middle cerebral artery (Figure 1d). The patient continued medical treatment showing considerable clinical improvement. After 1 month of medical treatment and about 10 days from the last MRI, she started complaining of nausea and had some episodes of vomiting again without any focal neurological deficits. These episodes were initially considered to be treatment-related and resolved with anti-emetic drugs. A follow-up MRI after 5 weeks of medical treatment, again to our surprise, showed a voluminous lesion in the right temporal lobe with midline shift, characterized by a dishomogenous signal intensity on *T*_2_ weighted (T2W) images, increased signal on unenhanced *T*_1_ weighted images (Figure 2a,b), and susceptibility changes on gradient-echo imaging. Similar cortical lesions with little perilesional edema were seen in the right frontal lobe (Figure 2a) and in the left temporo-mesial region. Both unenhanced phase-contrast and enhanced MR venography were performed that showed a right thrombosed transverse sinus and the vein of Labbè (Figure 2c, d). A diagnosis of multiple hemorrhagic cortical venous infarcts was made. The patient was now started on low molecular weight heparin (therapeutic dosage of enoxaparin 100 UI kg^–1^). With heparin the patient’s clinical status improved considerably. After 2 weeks of heparin therapy (and after 7 weeks of antitubercular therapy) a fourth follow-up MRI showed significant reduction in the size of all infarcts (Figure 2e) but revealed a new hypophyseal granulomatous lesion (Figure 2f) without pituitary symptoms. Endocrinologic tests were normal.

## Treatment

Treatment consisted of an intense initial phase (8 weeks) of four drug regimen (rifampicin, isoniazide, ethambutol, and pyranizamide) in our HIV negative patient with a non-drug-resistant strain of *M. tuberculosis*. The chronic continuation phase lasted 10 months with two drugs, isoniazide and rifamipicin. CNS localization of disease identified on MRI, prompted the addition of high-dose dexamethasone for 8 weeks as per international guidelines. Cortical venous infarcts were treated with subcutaneous injections of low-molecular weight heparin at a dose of 100 UI kg^–1^, two times a day for 3 months.

## Outcome and Follow-up

The patient was dismissed with antitubercular drug treatment for 1 year. A 1 year MRI follow-up showed no active lesions with complete resolution of sellar granuloma and venous infarcts. Also, her chest X-ray at 1 year follow-up was negative for active lesions and she is now off-therapy.

50% of patients with active TB have miliary disease on chest X-rays, and 1% will show CNS complications which is known to carry the highest mortality amongst all TB extrapulmonary complications.^3^ Focal neurological symptoms are rare but seizures, motor and cerebellar abnormalities are frequent. The virulence factor of the bacilli and the immune status of the patient are important factors in determining the clinical severity of CNS TB. In high-risk patients such as those living in endemic regions, immunocompromised, those who have recently been in contact with pulmonary TB and children must be screened for tuberculomas either with head CT or a brain MR.^2^

 The bacilli of *M. tuberculosis* reaches the CNS via a hematogenous route.^4, 5^ Rich et al first described a two-stage development of neurotuberculosis. Bacilli first localize in the brain (meninges, subpial, and subependymal surfaces) during bacteremia and remain dormant for years. Rupture of these foci (Rich’s focus) releases the bacilli into the subarachnoid space heralding the onset of meningitis.^6^ Our second patient probably had a two-stage development with rupture a Rich foci that led to multiple CNS localizations over a brief period of time.

MRI is a highly sensitive tool for detecting subtle meningeal complications, vasculitis and cranial nerve involvement. However, MRI may lack specificity failing to distinguish tuberculomas from other ring-enhancing lesions such as neurocysticercosis, toxoplasmosis or bacterial abscesses requiring additional tests. Miliary distribution of nodules in immunocompetent adults, elderly and those living in non-endemic regions can look very similar to metastatic disease. Primary lung, melanoma, and neuroectoderm tumors can spread via the hematogenous route and present miliary metastases.^7^ Whole body CT screening is warranted to search for primary tumor and possible multi-organ TB.

Tuberculomas are solid lesions (or tubercles) with or without a caseating necrotic core. These granulomas are surrounded by thick collagenous tissue and an outer layer of inflammatory mononuclear cells. The central core contains caseous necrosis. Outside the capsule variable degree of vasogenic edema and astrocytic proliferation has been reported.^8^ In both of our cases, numerous millimetric round lesions were seen characterized by increased signal on sequences with long repetition time (T2W and fluid-attenuated inversion-recovery images) surrounded by variable degree of edema without considerable mass effect. Solid thick complete ring enhancement was seen on contrast-enhanced images with a typical central hypointensity corresponding to necrotic core. The presence of a necrotic core depends on the size of the tuberculomas. Lesions too small may not show a ring-enhancing pattern.

TBM is characterized by a thick gelatinous exudate affecting the basal cisterns as seen in our second case. Abnormal leptomeningeal enhancement is more likely to be present over the cerebral convexities and sylvian fissures and less likely over the tentorium and the cerebellar meninges. However, absent or minimal meningeal enhancement may be seen in immuncompromised patients.^9^ Our patient presented with typical leptomeningeal enhancement at the level of the sylvian fissures and also around the optic chiasm and bilateral optic nerves. She also had a Rich focus at the level of the right sylvian fissure. It must be kept in mind that miliary form of CNS TB may not show obvious changes in CSF in the absence of meningeal involvement and such patients may remain clinically asymptomatic.^10^

Of the several CNS complications, obstructive communicating hydrocephalus carries the highest mortality.^11^ Obstructive communicating hydrocephalus is a direct consequence of leptomeningeal localization of *M. tuberculosis* hindering normal re-absorption of CSF through arachnoid granulations. Previous studies show 65% of patients with TBM will also have baseline hydrocephalus.^2, 11^ Our patient did not develop hydrocephalus.

Basal cistern exudates can cause inflammation of the vessel wall adventitia leading to their complete occlusion by reactive endothelial cellular proliferation and thrombus formation. Ischemic infarcts occur in 20–40% of patients with TB. It mostly affects the small lenticulostriate and thalamoperforating arteries.^12^ Most frequently, infarcts are seen in the basal ganglia and the internal capsule. Although rare, dural venous sinus and cortical venous thrombosis have been reported.^13^ These are possible complications of TBM and hypercoagulable state reported in patients with TB and can produce cortical hemorrhagic infarcts.^14^

 Although rare, TB-related granulomatous hypohysitis with peduncular thickening has been previously reported and can pose significant diagnostic challenge in the absence of other TB complications.^15^ Differential diagnosis includes Wegener’s granulomatosis, fungal infections, Langerhans cell histiocytosis or systemic autoimmune disorders.

## Learning points

1. Due to current demographic changes, TB is a now a worldwide problem.
2. CNS TB must be suspected in patients with non-specific neurological symptoms.
3. Serial MRI can be extremely valuable to allow modifications in treatment protocol and monitor evolution of CNS TB.
